# Nanotechnology-Based Strategies for Berberine Delivery System in Cancer Treatment: Pulling Strings to Keep Berberine in Power

**DOI:** 10.3389/fmolb.2020.624494

**Published:** 2021-01-15

**Authors:** Muhammad Javed Iqbal, Cristina Quispe, Zeeshan Javed, Haleema Sadia, Qamar Raza Qadri, Shahid Raza, Bahare Salehi, Natália Cruz-Martins, Zeinab Abdulwanis Mohamed, Mohammed Sani Jaafaru, Ahmad Faizal Abdull Razis, Javad Sharifi-Rad

**Affiliations:** ^1^Department of Biotechnology, Faculty of Sciences, University of Sialkot, Sialkot, Pakistan; ^2^Facultad de Ciencias de la Salud, Universidad Arturo Prat, Iquique, Chile; ^3^Lahore Garrison University, Lahore, Pakistan; ^4^Department of Biotechnology, BUITEMS, Quetta, Pakistan; ^5^Office of Research Innovation and Commercialization, Lahore Garrison University, Sector-C Phase VI, Defense Housing Authority (DHA), Lahore, Pakistan; ^6^Medical Ethics and Law Research Center, Shahid Beheshti University of Medical Sciences, Tehran, Iran; ^7^Department of Biomedicine, Faculty of Medicine, University of Porto, Porto, Portugal; ^8^Institute for Research and Innovation in Health (i3S), University of Porto, Porto, Portugal; ^9^Laboratory of Neuropsychophysiology, Faculty of Psychology and Education Sciences, University of Porto, Porto, Portugal; ^10^Laboratory of Molecular Biomedicine, Institute of Bioscience, Universiti Putra Malaysia, Serdang, Malaysia; ^11^Department of Biochemistry, Kaduna State University, Kaduna, Nigeria; ^12^Laboratory of Food Safety and Food Integrity, Institute of Tropical Agriculture and Food Security, Universiti Putra Malaysia, Serdang, Malaysia; ^13^Department of Food Science, Faculty of Food Science and Technology, Universiti Putra Malaysia, Serdang, Malaysia; ^14^Phytochemistry Research Center, Shahid Beheshti University of Medical Sciences, Tehran, Iran; ^15^Facultad de Medicina, Universidad del Azuay, Cuenca, Ecuador

**Keywords:** alkaloids, berberine, anticancer, nanodelivery systems, chemopreventive effects

## Abstract

Cancer is a multifactorial disease characterized by complex molecular landscape and altered cell pathways that results in an abnormal cell growth. Natural compounds are target-specific and pose a limited cytotoxicity; therefore, can aid in the development of new therapeutic interventions for the treatment of this versatile disease. Berberine is a member of the protoberberine alkaloids family, mainly present in the root, stem, and bark of various trees, and has a reputed anticancer activity. Nonetheless, the limited bioavailability and low absorption rate are the two major hindrances following berberine administration as only 0.5% of ingested berberine absorbed in small intestine while this percentage is further decreased to 0.35%, when enter in systemic circulation. Nano-based formulation is believed to be an ideal candidate to increase absorption percentage as at nano scale level, compounds can absorb rapidly in gut. Nanotechnology-based therapeutic approaches have been implemented to overcome such problems, ultimately promoting a higher efficacy in the treatment of a plethora of diseases. This review present and critically discusses the anti-proliferative role of berberine and the nanotechnology-based therapeutic strategies used for the nano-scale delivery of berberine. Finally, the current approaches and promising perspectives of latest delivery of this alkaloid are also critically analyzed and discussed.

## Introduction

Naturally-occurring bioactive compounds have gathered the attention of many scientists because of their limited cytotoxicity and high specificity (Salehi et al., 2019a). Indeed, plant-derived natural products are extraordinary reservoirs for the identification of various new drugs (Salehi et al., 2019a) with potent therapeutic abilities, used for centuries in traditional medicine for the treatment of different ailments (Atanasov et al., 2015; Marchese et al., 2016; Russo et al., 2016) due to their beneficial health effects (Wolfender et al., 2011; Cragg and Newman, 2013). Briefly, bioactive compounds derived from natural products are developed into either nutraceuticals or pharmaceuticals (Daliu et al., 2019; Munekata et al., 2020) and implemented for the treatment of various diseases for millennia (Sen and Chakraborty, 2017).

Cancer is a multifaceted disease orchestrated by a plethora of cellular processes and signaling cascades (Ahmad Farooqi et al., 2017). Considered a global health concern, the therapeutic options currently available for treating cancer are extremely lethal and provoke organs malfunctioning, which in turn reduces the life expectancy, besides to be extremely expensive (Hugtenburg et al., 2019). Natural products derivatives, such as phytochemicals, have emerged as potential chemopreventive agents able to reduce the risk of tumor initiation, metastasis, invasion, and spread (Zhao et al., 2018). Interestingly, a raising evidence have shown that phytochemicals share similar modes of action with conventional chemotherapeutic agents, and therefore, can be viewed as both adjuvants and therapeutic alternatives to the conventional antitumor therapies (Tiloke et al., 2018). In addition, when compared to synthetic chemopreventive agents, phytochemicals have limited cytotoxicity and their interplay with different molecular cascades makes them suitable anti-neoplastic agents (Neergheen et al., 2010).

Epidemiological studies have confirmed that the daily consumption of fruits, vegetables, plants, and herbal products can reduce the risk of several chronic diseases, such as diabetes mellitus (DM), cardiovascular (CV), and metabolic diseases, and even cancer (Hussain et al., 2016). Since plant-based products are rich in phytochemicals, their habitual intake improves the health status and prevent diseases (Manach et al., 2009).

Cancer is considered as the deadliest disease all over the globe. Many advance anti-cancer treatments are in the clinical and in pre-clinical trials to conquer the unbeatable fort of carcinogenesis. Natural (heterocyclic moieties) and synthetic products (Vinblastine and vincristine) are also in use as anticancer potential compound and it is believed to reduce the proliferation of the tumor cells, resulting in the increase of patient's survivals (Ali et al., 2015). Nano-based anticancer drugs have gained the spotlight of the world's scientific community, because of its targeted delivery drift. Nano medicines have a potential to deal with cancer even at an early stage with minimum side effects. These side effects can be eradicated by understanding the exact mode of action of these drugs (Ali, 2011). The fusion of metal complexes with thalidomide based Dicarbamate ligand has significant anti-cancer activity against Mcf-7 breast cancer cell lines. Copper, Nickle, and ruthenium ions were processed with ligand, and the resulting compound can be visualized by various spectroscopic techniques. Ligand and its complexes have strong binding affinity with DNA because of its high DNA binding constant ratio (Ali et al., 2013c). It is observed that curcumin ligand coupled with ruthenium ion has less toxicity to erythrocytes than the synthetic heterocyclic drugs currently used. This metal complex along with ligand has potential anticancer activities against HeLa, HepG2, MDA-MB-231, and HT-29 cell lines (Ali et al., 2013b). Imidazole is also reported as an anticancer agent and is not only an alkaloid and aromatic diazole, but also has high anticancer profile, because of its human friendly anticancer mechanism of action (Ali et al., 2017). Platinum compounds are also believed to be significant key player in coming times for the treatment of cancer. Although, these compounds are noted to have certain limitations, but platinum-based co-delivery can reduce these limitations. The third-generation platinum analogs (lobaplatin, heptalotin) are more superior than the second-generation analogs (carboplatin, oxaliplatin, nedaplatin) because of their better anticancer activities (Ali et al., 2013a). Certain drugs such as Salicin, naproxen, and diclofenac etc. are used to reduce the inflammation rate that is caused by cancer. All these anticancer drugs can be determined by certain chromatography techniques such as reverse phase liquid chromatography (RP-HPLC) and micro-HPLC (Sultan et al., 2005).

Berberine is an isoquinoline alkaloid derivative mostly obtained from the roots, stem, bark, and rhizomes of berberis, *Phellodendron amurense, Coptis chinesis*, and *Hydrastis canadensis* (Kong et al., 2004; Lee et al., 2012). These plants have been implemented in Chinese medicine for nearly 2,000 years for treating various diseases (Imenshahidi and Hosseinzadeh, 2016). However, the pharmacologically active component of these plants, i.e., berberine has only recently been identified (Imenshahidi and Hosseinzadeh, 2016). Berberine is a potent alkaloid with considerable pharmacological activities, including antimicrobial, antioxidant, anti-inflammatory, antidiabetic, hypoglycemic, hepatoprotective, and chemopreventive properties (Amritpal et al., 2010; Kumar et al., 2015). It is now clinically proven that berberine is a potential treatment for diseases, like diarrhea, ulcers, DM, CV diseases, hypercholesterolemia, fatty liver, polycystic ovary syndrome, and even cancer (Kumar et al., 2015; Imenshahidi and Hosseinzadeh, 2016). Regarding pharmacokinetics, berberine is not easily absorbed in the gut, despite it has been reported that berberine and its active metabolites are found to be higher in tissues as compared to blood following oral administration (Ye et al., 2009). Owing to these features, the delivery of berberine to target tissue site has become seriously challenging.

Berberine is one of the most used natural products Worldwide as more than 25 billion pills of berberine have been reported to used annually in Asia and African countries. It also has few limitations that it may cause intestinal side effects including cramping, stomach upset, and shaping gut microbiota. Pharmacokinetic profile of berberine has been reported in plenty of studies that it has low rate of absorption, especially when taken orally (Zhang et al., 2008; Alolga et al., 2016).

## Limitations of Berberine Usage

Extreme low absorption rate of bioactive berberine has been one of the biological pitfalls that hinder the use of berberine against multiple chronic disorders and cancer. It is documented that only 0.5% of ingested berberine absorbed in small intestine while this percentage is further decreased, when enter in systemic circulation. Different clinical trials have been noted to use as emulsifier enhancers to uplift the absorption rate of berberine in human to maximize its clinical efficacy. Different FDA approved food additives including TPGS, Quillaja extract etc. have been observed in clinical trials but nano-based formulation is believed to be an ideal candidate to increase absorption percentage as at nano scale level, compounds can absorb rapidly in gut and stability can be controlled via multiple characterization approaches (Sahibzada et al., 2018; Kwon et al., 2020).

Nanotechnological approaches for drug delivery have been put in practice for nearly a decade, with nanoformulations containing berberine appearing as a suitable therapeutic approach for several cancers (Mostafavi et al., 2019). In this sense, the present review presents comprehensive data on berberine chemistry, pharmacology, with special emphasis on its anticancer potential, and lastly provides an up-to-date vision on nanoformulations containing berberine as therapeutic/chemopreventive alternatives for both neoplastic and solid tumors.

## Chemistry

**Figure 1:**
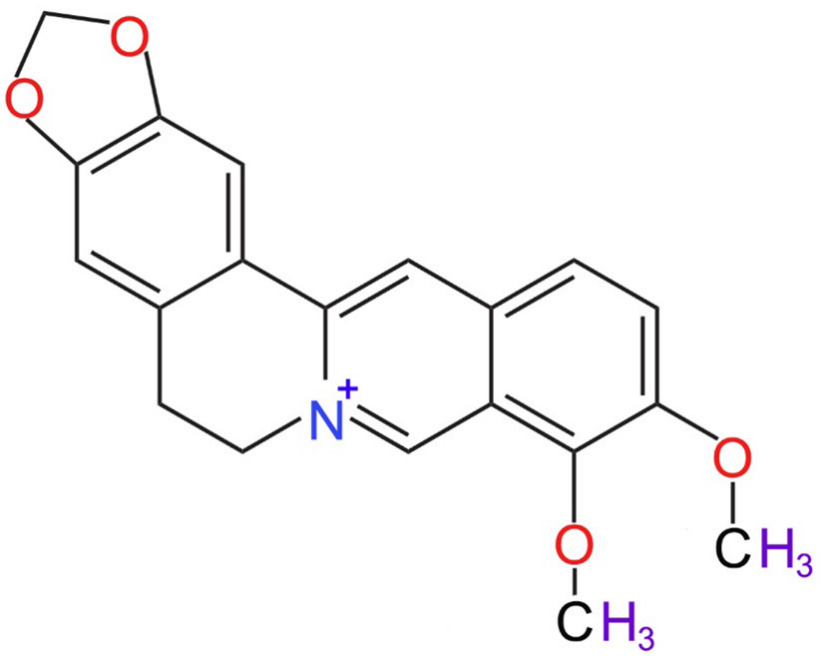
Chemical structure of berberine (National Center for Biotechnology Information, 2020).

Berberine, most specifically isoquinoline alkaloid berberine (Figure 1) is a member of the protoberberine alkaloids family (Kong et al., 2004). The most common member of this family are jatrorrhizine, columbamine, palmatine, coptisine, lambertine, canadine, and others (Grycová et al., 2007; Sun et al., 2009). The major sources of berberine includes barberry (*Berberis* spp.), meadow rue (*Thalictrum* spp.), celandine (*Chelidonium* spp.), goldenseal (*Hydrastis* spp.), and *Phellodendron* spp. (Singh and Sharma, 2018). Berberine is mostly isolated by alcoholic extraction (Fata et al., 2006), where following alcoholic extraction, the addition of acetic acid generates berberine chloride, hydrosulphate, and iodide berberine or dihydrodeoxyberberine (Fata et al., 2006).

Protoberberine alkaloids are widely recognized for their antimicrobial, anti-inflammatory, and antioxidant effects (Manske and Holmes, 2014). Specifically, plants belonging to the genus *Berberis* (barberry) have been implicated to in Japanese traditional medicine to treat cholera and diarrhea (Salehi et al., 2019b), while in Indian folk medicine, it has been used to treat cholera, malaria, and other gastric diseases (Salehi et al., 2019b). Several other reports have also shown that barberry can prevent metrorrhagia, renal bleeding, blood pressure reduction (Belwal et al., 2020), besides to be extensively used for the treatment of gastric disorders (Salehi et al., 2019b). The effects of barberry are mostly connected to the active compounds present in its chemical composition, namely berberine, where modifications of berberine have resulted in the production of several derivatives with efficient pharmacological properties (Table 1).

**Table 1:** Various physical properties of berberine (CAS 2086-83-1) (National Center for Biotechnology Information, 2020).

**Physical properties**	**Value**
Molecular weight	336.4 g/mol
Melting point	145°C
log *P*-value (octanol-water)	2.80
Atmospheric OH rate constant	2.09 × 10^−10^ cm^3^/molecule-sec at 25°C

## Berberine as Anticancer Drug

Berberine has begun to emerge as a potent anticancer agent over the years (Sun et al., 2009; Liu et al., 2019). Berberine has tremendous antioxidant effects, which makes it suitable for the treatment of various diseases, such as DM, diarrhea, hormonal disorders, and so on (Tillhon et al., 2012). However, the pro-apoptotic role of this alkaloid was bleak. Berberine has been reported to inhibit proliferation, metastasis in various cancers, such as leukemia, colorectal, prostate, lung, glioma, and ovarian cancers (Jantova et al., 2007; Chen et al., 2015; Guamán Ortiz et al., 2015; Lu et al., 2016; Tian et al., 2016; Wang et al., 2016), although there are certain hindrances which have greatly affected the tumor inhibitory properties of berberine. The major reason for this limited apoptotic role is its poor absorption and limited bioactivity (Chen et al., 2014). However, with the advancements in the field of nanotechnology these issues can be overcome.

Berberine exerts its apoptotic role in tumor cells via activation of pro-apoptotic genes. Briefly, berberine is able to disrupt the B-cell Lymphoma-2/Bcl-2 associated protein X (Bcl-2/Bax) ratio, which in turn decreases the mitochondrial membrane potential of tumor cells (Li et al., 2017). It also triggers the caspases activation through intrinsic apoptotic pathway, consequently, it activates caspase-3 and−8 which release cytochrome C (Li et al., 2018). In addition, berberine is also an activator and modulator of cell cycle; specifically, it influences the cell cycle activity via inducing cell cycle arrest in tumor cells at the G1 phase. Interestingly, at lower concentrations, berberine inhibits tumor cells growth at G1 phase, while at higher concentrations it triggers growth arrest at G2/M phase of cell cycle (Eo et al., 2015). Moreover, it has been reported that berberine promote Reactive Oxygen Species (ROS) production in tumor cells, ultimately triggering apoptosis. Other studies have reported that the combination of chemotherapy and berberine can reduce the cytotoxic effects of these drugs and improve their therapeutic efficacy, thus, enhancing the therapeutic outcomes in various cancers (Park et al., 2015).

Being a natural compound, berberine has limited cytotoxicity. A number of studies have shed light on the fact that berberine is extremely target-specific and induces apoptosis in tumor cells with limited cytotoxicity on normal cells (Liu et al., 2011; Yang and Huang, 2013). However, the low absorption rate and poor stability are the two major stumbling blocks, which have reduced its cytoprotective efficacy (Liu et al., 2016). Consequently, new approaches toward stabilizing berberine and increasing it absorption have been made. This has led toward the development of novel berberine derivatives with changes at different structural positions, such as the C8, C9, C10, C12, and C13 for improved stability and biological activity (Milata et al., 2019). These modifications have been reported to increase the antimicrobial activity of berberine (Milata et al., 2019), although the change at C9 position has been evidenced to increase the antitumor potential of berberine (Zou et al., 2017). Altogether these findings indicate that berberine is a potentially suitable drug for chemoprevention and therapeutic interventions.

## Bioformulation of Nanoparticles as Berberine Carrier

Berberine has been widely used as potential therapeutic agent in Traditional Chinese and herbal medicines to address multiple biological issues, including cancer. In modern medicine, berberine is classified under class III drug as per biopharmaceutical classification system (BCS) due to its poor membrane permeability. In this way, nanotechnology has opened a new avenue for novel development of drug delivery vehicles to improve cancer treatment. Indeed, nanodelivery strategies of anti-cancer drugs have been considered an iron gate for cancer therapy. However, to obtain optimal benefit from this potential biological compound, modern biotechnology have offered different nanoencapsulation approaches, including magnetic nanoparticles, semi-crystalline nanoparticles, phosphor-lipid drug carriers, silver nanoparticles, mitochondrial targeted nanocarrier, alkylated berberine, berberine-loaded liposomes, pH sensitive lipid-base nanocarriers, electrochemical DNA biosensor, chitosan nanoparticles, niosome and lipo-niosome formulations, to enhance berberine delivery and bioavailability in the targeted regions of body. Thus, nanoformulations containing berberine have revealed to be extremely efficient in enhancing berberine stability as well as its oral availability. In the next sub-sections, we discuss in detail the nanotechnology-based therapeutic interventions of berberine and their role in treating cancer.

Many herbal drugs have been used with different combinations to enhance anticancer effect with minimize side effects (Ali et al., 2019b). *In vitro* analysis was performed by using chloroform extract of clove bud on H1299 and A549 human cancer cell lines to study cellular migration and apoptosis. It was noted that natural compound of clove extract possesses inhibitory effects on cellular migration and induce apoptosis. It is believed that clove bud extract could be significant contributing agent for lung cancer treatment and its effectiveness can be improve by nano-formulation approach to enhance its bioavailability (Ali et al., 2019a).

### Nanotechnology Applied to Berberine: An Overview

Nanotechnology and nanomedicines have gain huge acceptance among scientific community and to what concerns to berberine, there is need to formulate bio-composition with berberine at nano-level to get maximum benefit from traditional therapy. The therapeutic efficacy of any molecule is directly proportional to its solubility, and in pharmaceutical industry solubility is one of the key factors that can affect drug pharmacokinetics (Cheng et al., 2010; Mullauer et al., 2011). One of the approaches to address this trouble is “particle size reduction,” in which drug molecules are converted into nanosize delivery agents. Both bottom up and top down approach have been frequently used in pharmaceutical industry, with nanomaterials being synthesized by physical, chemical, and biological methods or by combining any all methodologies (Table 2).

**Table 2:** Most common approaches to synthesize nanomaterials as drug carrier.

**Physical**	**Chemical**	**Biological**
**METHODOLOGIES FOR SYNTHESIS OF NANOMATERIALS**		
High energy ball milling	Sol-gel synthesis	Microorganism assisted biogenesis
Pulse vapor deposition	Micro-emulsion	Bio-template assisted biogenesis
Laser pyrolysis	Hydrothermal synthesis	Plant extract assisted biogenesis
Flash spray pyrolysis	Polyol-synthesis	
Electro-spraying	Chemical vapor synthesis	
Electro-spinning	Plasma enhanced chemical vapor deposition	
Melt mixing		

Physical approach involves mechanical pressure, high energy radiation, magnetic field, electro thermal, and different forces to synthesize nanomaterials. Chemical methodology also relies upon top-down and bottom up methodology, involving a chemical reagent to stimulate nanomaterial production. Both physical and chemical strategies are not much cost effective and require the involvement of biological entities to make it more system friendly and feasible. Biological synthesis makes use of biosystems, like bacteria, viruses, yeasts, actinomycetes, and a variety of potential plant extract and enzymes (Table 2). Biological synthesis of nanomaterials has been proved to be more effective strategy for biomaterial synthesis for clinical application as biological compound act as capping and oxidizing agents to strengthen the cytoprotective ability for healthy cells and maximize biological stability for cellular micro-environment (Iqbal et al., 2018).

One of the simplest methods to enhance berberine efficiency as anti-cancer agent is the conversion of crystalline structure into semi-crystalline by evaporative precipitation of nano-suspensions (EPN) and anti-solvent precipitation with syringe pump (APSP) approaches. It is observed that there is significant rise in solubility and dissolution rate (Sahibzada et al., 2018). Antisolvent precipitation is one of the easy, affordable, and feasible approaches that base of solvent displacement method for rapid nano-suspension preparation (Bilati et al., 2005). Hypromellose and propylene glycol could be used as stabilizers in both approaches. In EPN, pure drug saturated solution is prepared in ethanol and by rapid addition of hexane as anti-solvent, to form nano-suspension. Nano size drug particles are obtained by rapid evaporation under vacuum by using rotary evaporator followed by vacuum drying of nanoparticles to evaporate all solvents and antisolvents (Kakran et al., 2012). In APSP, ethanol is used as solvent to prepare a saturated solution of berberine, and deionized water is added as anti-solvent by rapid injecting approach using syringe under constant mechanical stirrer. The solution is subjected to rotary evaporator to get nano-sized drug particles (Kakran et al., 2012; Sahibzada et al., 2018).

### Berberine Loaded Solid Nanoparticles

A folate acid modified chitosan nanoparticle loaded berberine hydrochloride (BH/FA-CTS NPs) was synthesized using ion cross linkage technique. These nanocomposites have proved to be new players to treat cancer by regulating apoptosis and inhibiting Cellosaurus cell line-1 (CNE-1) cells migration and proliferation (Wang et al., 2018).

Lyotropic liquid crystalline nanoparticles (LCNs) have been used by researchers to treat breast cancer progression and to enhance the berberine solubility. LCNs nanoparticles are synthesized by ultra-sonication method, using monoolein, PEG, poloxamer, and transcutol. LCN are reported as a potential drug nanocarrier to enhance the cell uptake of berberine in Caco-2 cells (Loo et al., 2020).

Carcinoma of squamous cells and liver badly threaten human life. It is seemed that berberine has anti-hepatocarcinoma and anti-esophageal carcinoma properties. In a study, berberine delivered to a patient body through solid lipid nanoparticles, prepared through pressure homogenization technique, revealed inhibitory effects on the proliferation of HepG, Huh7 and EC9706 cells by MTT assay (Meng et al., 2016).

**Figure 2:**
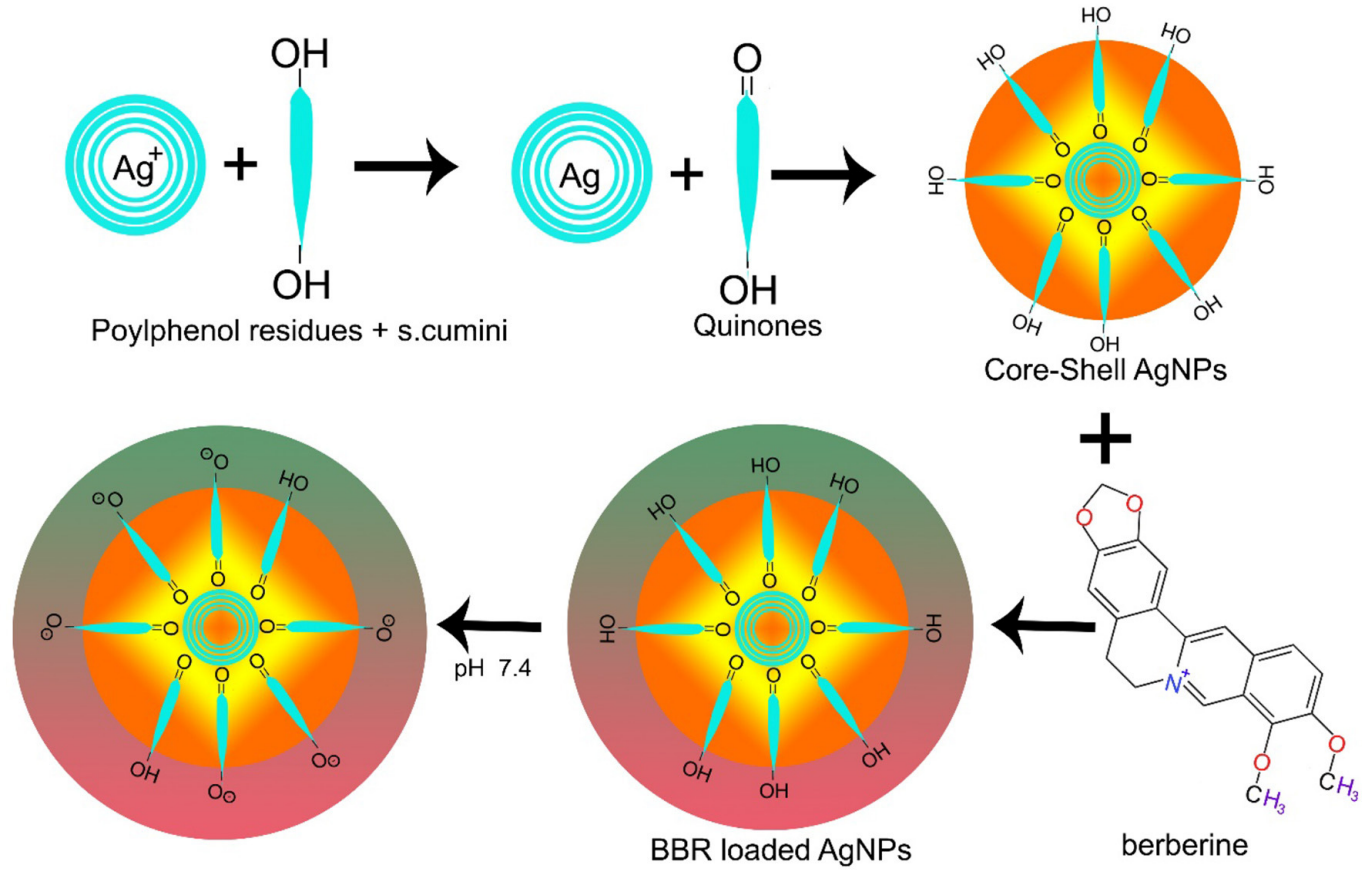
Synthesis of BBR loaded silver nanoparticles.

AgNPs have also been used in combination with other materials. FA-PEG@BBR-AgNPs have been reported of being used to treat breast cancer, ultimately overcoming the considerable toxic effects caused by chemotherapeutic drugs. FA-PEG@BBR-AgNPs are formed when citrate crowned silver nanoparticles encapsulate berberine with polyethylene glycol (PEG) containing folic acid, leading to the systematic delivery of BBR into targeted cancerous site (Figure 2). In this particular formulation, PEG are used to overcome drug toxic effects, while folic acid triggers the receptor-mediated endocytosis (Bhanumathi et al., 2018).

**Figure 3:**
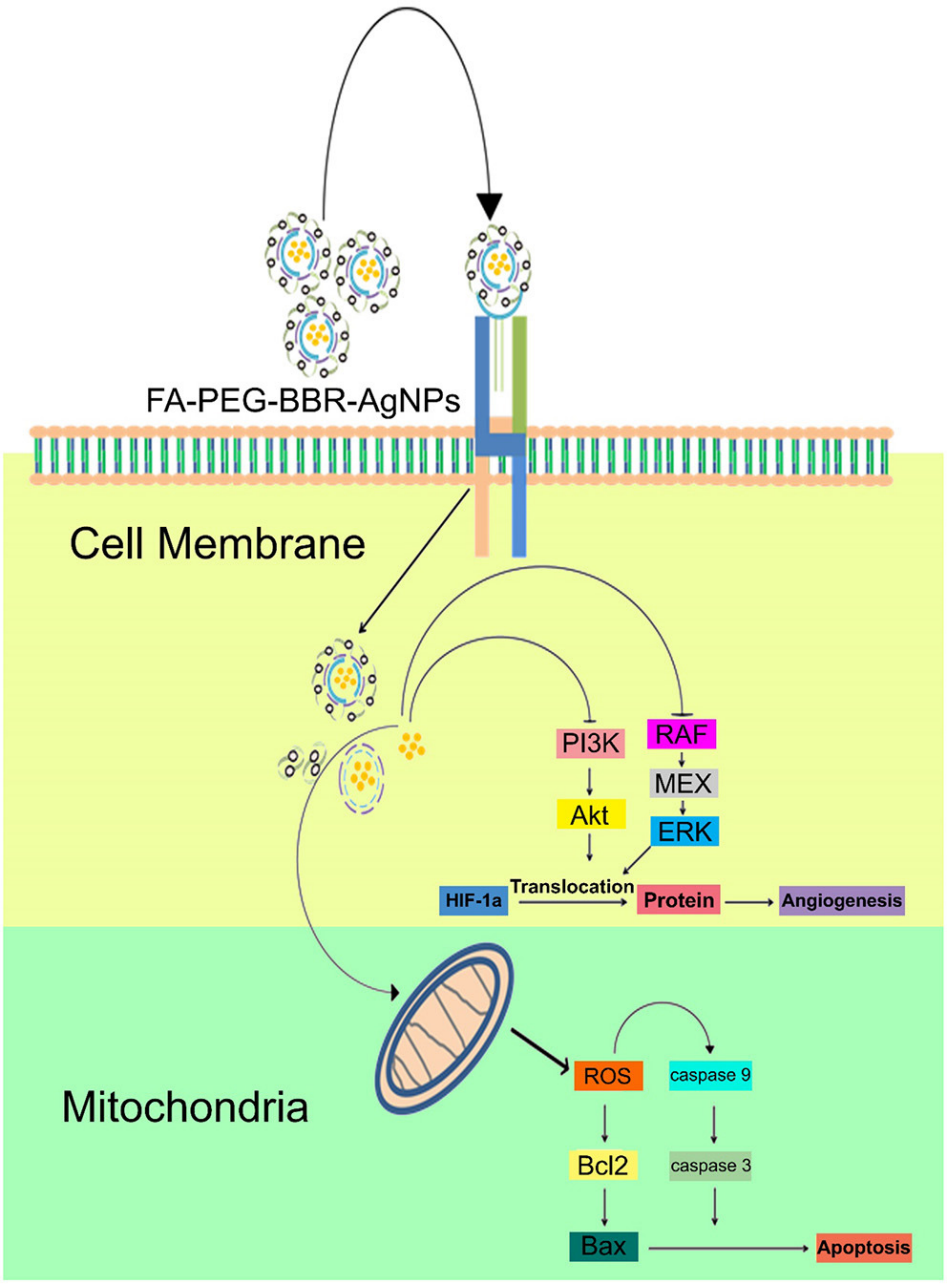
Mechanism of action of FA-PEG-BBR-AgNPs in breast cancer cells.

In breast cancer cells, nanomaterials disassemble into their constituents (BBR and AgNPs) in the cell's cytoplasm, resulting in the downregulation of PI3K/AKT and Ras/Raf/ERK to hinder HIF-1α expression (Figure 3) (Wang et al., 2017a; Bhanumathi et al., 2018).

A recent study highlighted the application of lipid-based nanoparticles as berberine nano-biocarriers to enhance its bioavailability and to limit the aggressive cell division in B16F10 melanoma cells. The *in vivo* therapeutic effect of berberine-loaded lipid nanoparticles was also assessed on C57BL/6 metastasis tumor mice. Briefly, C56BL/6 is an inbred strain of laboratory mice, a commonly used a model organism for human disease genetic experimentation and analysis, while B16F10 is the murine cancer cell line derived from C56BL/6 cancer model mice. *In vivo* and *in vitro* findings revealed that the lipid-based nanoparticles approach to deliver berberine and other drug compounds is an interesting strategy to limit the aggressive metastatic tumor. Indeed, a raising number of scientific data have highlighted the use of such approach on leukemia, hepatoma, breast, lung, and prostate cancer, among others, besides to be target of an exceptional attention in cancer research (Parhi et al., 2017; Guo et al., 2019).

### Magnetic Nanoparticles as Berberine Nanocarriers

Magnetic nanoparticles hold a great promise for targeted and controlled drug delivery system under the influence of magnetic field. Iron oxide nanomaterials with different formulations have been reported to enhance drug efficacy and control cancer cell progression in model organisms. Iron oxide nanoparticles are considered as ideal candidates for site specific drug delivery system due to its biocompatibility and magnetic properties. Codelivery study of tumor regression was performed by injecting iron oxide nanoparticles and berberine complex (FeO-BBR) in the hind limbs of mice with solid tumor. Oral administration of FeO-BBR complex in different *in vivo* analysis on mice models, directed by magnetic field, proved to be a potential strategy to reduce tumor diameter and trigger the biological process of apoptosis. Histopathological examination suggest that these biocompatible nano-drug complexes have no impact on surrounded normal cells (Sreeja and Nair, 2015; Yang et al., 2016). Drug-loaded magnetic nanoparticles prepared by co-precipitation methodology were also addressed on prostate cancer DU145 cells. It was found that the codelivery of anticancer drug with magnetic particles inhibit the cancer cells proliferation. Gene expression analysis by Comet assay and qRT-PCR established a molecular relation, consisting of changes in the expression level of bax, bcl, and trigger caspases activity to induce apoptosis (Lee et al., 2013; Sreeja and Nair, 2015).

Janus magnetic mesoporous silica nanoparticles (Fe_3_O_4_-mSiO_2_NPs) have also been developed for berberine delivery. Structurally, Fe_3_O_4_-mSiO_2_NPs have SiO_2_ body and Fe_3_O_4_ head for magnetic targeting. To develop a tumor microenvironment responsive nanoparticle with uniform morphology, good superparamagnetic properties, high drug-loading amounts, superior endocytic ability, and low cytotoxicity, a pH sensitive group is reported to be attached on mesoporous silica. Due to this pH sensitive group, nanoparticles have high endocytic activity in hepatocarcinoma cells. Berberine was pre-loaded into folic acid targeting Janus gold mesoporous silica nanocarriers (FA-JGMSNs), capable of overcoming the limited solubility of berberine. Data obtained from *in vitro* and *in vivo* studies indicate that these nanoparticles have an effective antitumor activity (Wang et al., 2017a,b; Li et al., 2019).

Additionally, hypoxia-specific chemo-targeting iron-oxide nanoparticles containing berberine complexes have been used in mouse models to control cancer proliferation. In a study, NP-BBN-SAN complexes were injected in mice with hind limb tumor. As main findings, nano-complex revealed to be a significant agent to reduce tumor diameter by inducing bax and caspase activity and declining the expression of akt and bcl2 genes in tumor microenvironment, involved in apoptosis upregulation. SAN accumulates in the hypoxic area of tumor, targeting cytotoxic drug into the tumor area which further undergoes tumor regression (Sreeja and Nair, 2018). Thus, the combination of SAN and berberine nanoparticles proved to be a potential target to raise the therapeutic efficacy against different cancers.

The multiple drug resistant (MDR) behavior of cancer cells limits the effectivity of anticancer chemotherapeutics. Niosomes have been reported to be used in multiple research trials to address MDR complex cell response, as niosome nanoparticles are nanosized non-ionic surfactant-based vehicle in aqueous media that have more intracellular penetration capacity (Tavano et al., 2014). A niosome formulation of surfactant and cholesterol offer a combined therapy platform and has been used as a drug delivery carrier for anticancer model drugs, including 5-fluorouracil (5-FU) and doxorubicin. In this formulation, the bioactive compounds, curcumin and berberine, have been reported as chemosensitizers and are potential agents to reduce MDR cell behavior. More recently, a new liponiosome formulation is viewed as an emerging codelivery nanocarrier approach, formulated by the combination of niosome and liposomes. Liponiosome offers pH sensitive control drug release and increased entrapment percentage in cancer cells against Saos-2, MG-63, MCF-7, and K-1 cell lines (Tavano et al., 2014; Naderinezhad et al., 2017). Thus, the codelivery of anti-cancer drugs with bio-based nanosized functional nanoparticles provide a unique platform to address MDR-related issues for multiple cancer treatments.

Hydrophobin (HFB-1) coated nanoscale formulation of niosome loaded with anti-cancer drugs has also been recently reported as a potential candidate for effective and selective drug delivery systems. The coating of nano-drug delivery systems with fungal spore protein HFB-1 promotes *in vivo* immune system identification by inhibiting immune response and have been considered a novel strategy with increased effectivity against different cancer cell lines (Barani et al., 2020). In addition, and also worth of note is that, a large number of cancer-associated infections have been reported throughout the globe, including stomach, oral, lung, colon, and gall bladder carcinoma and prostate cancers. In such affections, a niosomal formulation, carried out by using tween 60, cholesterol and span 60, and using deoxycycline as a potential antibiotic to treat infection-related cancers, was prepared. The synthetized deoxycycline-loaded niosomes were assessed for *in vitro* antimicrobial and anticancer activity. As mains findings, the authors stated an enhanced potential using the niosomal formulation, and found a profound chemotherapeutic effect against prostate cancer (PC3) cells (Akbarzadeh et al., 2020).

### Silver Nanoparticles as Berberine Nanocarriers

Silver nanoparticles are also being used as carriers for berberine delivery to target site. Recent data have supported that the anticancer activity of berberine is linked to the downregulation of adenosine monophosphate activated protein kinase (AMPK) and hypoxia-inducible factor 1-alpha (HIF-1α), inhibition of cell proliferation, apoptosis induction, angiogenesis arrest, and suppression of malignant growths (Mirhadi et al., 2018). In addition, and regarding the most often studied cell lines, MCF-7 cell lines are seen to be the more sensitive to berberine-loaded silver nanoparticles. Thus, in a xenografted mice model, berberine-loaded silver nanoparticles were able to decrease the tumor volume and weight by inhibiting the MCF-7 cells proliferation, without reducing the body weight of mice model (Bhanumathi et al., 2017). Moreover, in a mice model with breast cancer, berberine loaded AgNPs were able to efficiently enter and disassemble the breast cancer cells cytoplasm to release its contents, i.e., nanoparticles and berberine, thereby, inducing cytotoxic effects and triggering apoptosis by hampering HIF-1α expression, through inhibition of PI3K/AKT and Ras/Raf/ERK proteins expression in signaling pathways, and generating ROS, respectively (Bhanumathi et al., 2018).

### Chitosan-Based Nanoparticles as Berberine Nanocarriers

Chitosan-based nanoparticles, due to their ability of improving berberine bioavailability, also act as a berberine delivery vehicle to target tumor cells. Berberine hydrochloride loaded chitosan-based nanoparticles are reported to induce apoptosis in nasopharyngeal carcinoma epithelioid cell line (CNE-1) through FRs-mediated endocytosis pathway (Wang et al., 2018).

Doxorubicin is the modern anticancer antibiotic with molecular DNA intercalation ability, approved by the Food and Drug Administration (FDA) for multidrug-resistance cancer treatment in PEG-Liposomal formulation (Doxil). To enhance doxorubicin efficacy and cell permeability, Dox-loaded nanocarriers have been considered one of the most promising strategies to suppress cancer cell progression (Schroeder et al., 2012).

Mitochondria targeting nano-drug delivery agents are designed to invade the cancer cell mitochondrial membrane by getting benefit from the increase mitochondrial membrane potential of cancer cells (Fu et al., 2015; Tuo et al., 2016). Recent studies have highlighted the use and development of berberine-mediated mitochondrial targeting nanodelivery system for various *in vitro, in vivo* and *ex vivo* experiments against multiple drug resistant cancer cells. One of the optimal strategies is to target cancer cell mitochondria by the delivery of chemotherapeutic agents, including doxorubicin, to limit the uncontrolled cells proliferation. One of the illustrative examples comes from the studies to enhance doxorubicin uptake; briefly, in such studies, alkylated berberine is reported to act as a mitochondrial targeting ligand, but derivatives of alkylated berberine with reported modification at C9th and C13th positions are proved to be more effective in targeting cancer cell mitochondria than berberine alone (Weissig and Torchilin, 2001; Tuo et al., 2016). The same study, performed with further fine modifications, with consist in the addition of alkyl chain in liposome bilayer membrane, a 16 carbon aliphatic chain was introduced into the C 9th position of berberine “9-C16 BBR” to synthesize mitochondrial targeting doxorubicin loading folic acid conjugated polyethylene glycol liposome (MT-Dox-FOL-PEG-liposomes). The synergistic delivery of this novel alkylated berberine proved to induce significant apoptosis and cytotoxic activity in multiple drug resistant MCF-7 and adr cells when compared to the combined regular delivery of doxorubicin and liposomes. This nano mitochondrial targeting agent “MT-Dox-FOL-PEG-liposomes” increased cell permeability up to 15 folds in *in vitro* examination. Additionally, *in vivo* and *ex vivo* studies in drug resistant MCF-7/Adr mice xenograft cells was performed and “MT-Dox-FOL-PEG-liposomes” proved to be a potential candidate for tumor proliferation inhibition (Pereira et al., 2007; Tuo et al., 2016).

### DNA Nano-Biosensors to Determine the Effect of Berberine on Cancer Cells

Multilayer nanofilms are getting increased acceptance as ideal candidates for the controlled and targeted systemic release of anticancer drugs. Programmable 3D DNA origami structures have been designed by layer deposition methodology. They have a unique functional nano size surface architecture and proved to have a profound impact to increase the drug release profile (Cho et al., 2014).

Nano-biosensors have been used for early stage detection of various genetic, cancer, and harmful diseases. They are considered fast, simple, and cost-effective approaches for disease diagnosis. Several types of DNA-based nano-biosensors have been used to assist the effective delivery of personalized medicine (Abu-Salah et al., 2015). Recently, quantum dot-based DNA nano-sensors have been used to detect the DNA at low concentration, based on fluorescence resonance energy transfer. Quantum dots are reported to act as a concentrator which amplify the target signal and are used in point mutations detection (Hu et al., 2017). Moreover, AuDE/CYS/rGO/AuNP/dsDNA nano-biosensors have also been employed along with surface-enhanced Raman spectroscopy (SERS)/electrochemical transduction to detect the effect of anticancer drugs on DNA. DNA nano-biosensors have the potential of drug screening by monitoring the DNA modification or DNA damage induced by a specific anticancer agent. Thus, they proved to be a beneficial approach for the development of anticancer therapies. In these nano-biosensors, the intercalation of anticancer drug on DNA is identified through SERS signals, and the drug-dose efficacy is evaluated through electrochemical signals (Ilkhani et al., 2016).

Double stranded DNA nano-biosensors composed of multi-walled CNTs (MWNTs), colloidal gold nanoparticles (GNPs), and GNP-MWNT mixture in different solutions, like dimethyl formamide, sodium dodecyl sulfate, and phosphate buffer, respectively, has also been used to investigate the effect of berberine on cancer cells. Through nano biosensors, it was observed that berberine triggers DNA fragmentation in U937 cancer cells by inducing structural changes in DNA. Berberine also revealed to be able to intercalate with cancer cells DNA and cause strand breaks followed by opening of DNA helical structure (Ovádeková et al., 2006).

Extensive studies have been performed to understand the possible molecular mechanisms through which berberine induce its anticancer effects in biological system. For example, an *in vivo* investigation performed in rats with colon cancer decipher that berberine markedly inhibit cell proliferation. At cellular level, berberine trigger cell death by inhibiting mRNA expression of β-catenin in colon cancer cells, linked to Wnt/β-catenin signaling cascade (Wu et al., 2012). Berberine is also reported to induce auto-phagocytosis and inhibit cell proliferation in human cancer HepG2 and MHCC97 cell lines. Molecular evidence has also shown that berberine induce bax-mediated apoptosis by stimulating caspase 3/9 activity. Molecular interaction of berberine is also reported to induce the Atg-5 pro-apoptotic and autophagy gene. There is ample evidence from scientific research that berberine promotes upregulation of Beclin-1 and downregulation of mTOR gene. Worth of note is that the upregulation of MAP-Kinase signaling cascade and p53 gene are the key molecular process to promote autophagy in cancer cells (Wang et al., 2010; Zhao et al., 2019; Kim et al., 2020).

## Conclusion and Upcoming Perspectives

Berberine is an isoquinoline alkaloid that has been used from thousands of years for multiple health affections. In recent years, the shifting pendulum of therapeutics for naturally-occurring anticancer molecules have been hub of research. Indeed, traditional herbal products, including berberine, berbamine, Ginkgo Biloba, curcumin, quercetin have been reported to have a great impact on tumor metastasis through several pre-clinical and clinical studies (Sithranga Boopathy and Kathiresan, 2010; Cragg and Newman, 2013). Indeed, berberine, as well as many other natural products, may serve as an iron gate for cancer therapy and have shown to be effective against multiple types of cancer, although its limited cell permeability and dissolution rate due to brief plasma half-life have hampered its effective use. Thus, to enhance the anti-metastatic and anti-tumor efficacy of berberine, nanobiotechnology have offered a nano-platform to solve this big trouble by nanodelivery bioformulation approach.

Nanomedicines with unique technological improved version would be the future of pharmaceutics in this rapid changing World. The breakthrough in computational biology and artificial intelligence are believed to play a decisive role to precisely target the culprit cancer causing cells in our biological system. Particle size reduction approach to increase bioavailability of green compounds and the biological activity of soluble nano drug systems are gaining huge acceptance in clinical traits. Currently available data have shown that berberine delivery via nanocarriers in specific cancer sites are reported to upregulate p53, bax, beclin-1, caspases activity, including caspase3/9, MAPK signaling cascade and to downregulate the expression of Bcl-2, mTOR, Wnt/β-catenin. However, despite the berberine bioconjugation and bioformulation with potential agents to enhance its efficacy have emerged as therapeutic demand to step forward to conquer the unbeatable fort of cancer progression, further studies are still needed to support such health effects and upcoming medical applications.

Herbal bioactive compounds are reported to have few pharmacokinetic limitations including poor aqueous solubility, low bioavailability and less effectiveness, low site-specific delivery, and stability concern that demand for novel formulations to overcome disadvantages of natural products. Nanoplatform offers multiple codelivery strategies of berberine with other anti-cancer drugs and to enhance stability of natural compounds by preventing from low pH and metabolic effects in gut that prolong its stability to remain in bloodstream and enhance bioavailability (Mirhadi et al., 2018). Cellular toxicity and unwanted side-effects of targeted nano-herbal formulations is limited by minimizing the toxic level dose. Huge published data from the literature support the non-toxic impact of multiple nanomedicines (Voigt et al., 2014). However few nanomaterials are also reported in the literature to enhance cellular toxicity and oxidative stress including CNT, fullerene, and metal oxide (Bonner, 2007). Such hinderance in the clinical application of nano-nutraceuticals formulations and diagnosis are now resolved by using selective nano-materials for clinical purposes as cerium oxide based nano-materials are reported have anti-oxidant potential an many other magnetic nanoparticles are use in MRI and FMIR diagnosis procedures with no or limited side effects (Dhall and Self, 2018).

Optical and magnetic properties of nano-carriers conjugated with drug and targeted moieties would be suitable candidate to address the toxicity challenges significantly. Hybrid nano-structures are expected to strengthen the application of nanomedicines more effectively in future. Hybrid nanomaterials of unique elements like cerium with multiple oxidation states and pseudo infinite half-life could serve as potential addition in pharmaceutical industry to limit cancer recurrence and metastasis (Xu et al., 2020).

## Author Contributions

MJ, ZJ, HS, QQ, SR, and JS-R: conceptualization. All authors: validation investigation – data curation writing, read, and approved the final manuscript. ZA, ZJ, CQ, MS, SR, BS, NC-M, AA, and JS-R: review and editing.

## Conflict of Interest

The authors declare that the research was conducted in the absence of any commercial or financial relationships that could be construed as a potential conflict of interest.

## Abbreviations

Bcl-2/Bax: B-cell lymphoma-2/Bcl-2 associated protein X
ROS: Reactive Oxygen species
BCS: Biopharmaceutical classification system
EPN: Evaporative Precipitation of nano-suspensions
APSP: anti-solvent precipitation with syringe pump
BH/FA-CTS NPs: folate acid modified chitosan nanoparticle loaded berberine hydrochloride
CNE-1: Cellosaurus cell line-1
LCNs: Lyotropic liquid crystalline nanoparticles
PEG: polyethylene glycol
PI3K/AKT: Phosphatidylinositol-3-Kinase and Protein Kinase B
Ras: Rapidly Accelerated Sarcoma
Raf: Rapidly Accelerated Fibrosarcoma
ERK: Extracellular Receptor Kinase
HIF-1α: Hypoxia inducing factor 1-alpha
FA-JGMSNs: Janus gold mesoporous silica nanocarriers
MDR: Multiple Drug Resistant
HFB-1: Hydrophobin
AMPK: Adenosine Monophosphate Activated Protein Kinase
MT-Dox-FOL-PEG-liposomes: Mitochondrial Targeting Doxorubicin loading Folic Acid Conjugated Polyethylene glycol liposome
MAPK: mitogen activated protein kinase
mTOR: Mammalian target of Rapamycin.
